# Assembling and validating a heart failure-free cohort from the Reasons for Geographic and Racial Differences in Stroke (REGARDS) study

**DOI:** 10.1186/s12874-019-0890-x

**Published:** 2020-03-04

**Authors:** Parag Goyal, Matthew T. Mefford, Ligong Chen, Madeline R. Sterling, Raegan W. Durant, Monika M. Safford, Emily B. Levitan

**Affiliations:** 1 Department of Medicine, Weill Cornell Medicine, New York, NY USA; 2 Department of Epidemiology, University of Alabama at Birmingham, Birmingham, AL USA; 3 Department of Medicine, University of Alabama at Birmingham, Birmingham, AL USA

**Keywords:** Heart failure, Epidemiology, Population studies

## Abstract

**Background:**

Studies examining incident heart failure (HF) have been limited to select populations. To examine incident HF with broader generalizability, there is need to assemble a HF-free cohort using a geographically-diverse sample. We aimed to develop and validate a simple medication-based strategy for assembling a HF-free cohort from the REasons for Geographic And Racial Differences in Stroke (REGARDS) study.

**Methods:**

We examined REGARDS participants with ≥6 months of Medicare inpatient and outpatient claims data at the time of the baseline in-home study examination. To assemble a HF-free cohort, we identified and excluded participants taking HF-specific medications. To validate this approach, we evaluated event rates among this cohort and assessed diagnostic performance using Medicare claims-based definitions of HF as the referent standard.

**Results:**

Among 28,884 eligible participants, 3125 were excluded from the proposed HF-free cohort, leaving a total of 25,759 (89%) participants. Depending on the Medicare definition used as the referent, the negative predictive value of this approach ranged from 95.0–99.2%. Negative predictive value was stable across age, sex, and race strata.

**Conclusions:**

The approach to assemble a HF-free cohort in REGARDS can serve as the basis for future studies to examine incident HF in REGARDS and similar studies.

## Background

Approximately 6 million people live with heart failure (HF) in the United States [1]. Understanding features of incident HF, such as risk factors for its development, can inform prevention and screening efforts and thus mitigate the effects of the HF epidemic. Studies to date examining incident HF have provided insight into key features, but have been limited to select populations. For example, reports from community-based cohorts in Olmstead County, Minnesota [2] and Framingham, Massachusetts [3] have described predominantly White populations; and reports from randomized-controlled trials have described populations that meet strict inclusion criteria, excluding many older adults and individuals with comorbidities [4, 5]. While studies from multicenter observational cohorts like the Cardiovascular Health Study (CHS), [6] Dynamics of Health, Aging, and Body Composition study (Health ABC), [7] and the Atherosclerosis Risk in Communities study (ARIC) [8] have reported on more racially and ethnically diverse populations, study subjects originated from select communities which do not necessarily reflect the rest of the United States. Consequently, there is a need to examine incident HF using a contemporary racially and geographically diverse United States cohort. The REasons for Geographic And Racial Differences in Stroke (REGARDS) study [9] is a cohort of over 30,000 community-dwelling African American and white adults from across the 48 contiguous United States, and is thus larger than CHS and Health ABC, and more geographically-diverse than ARIC. With a rigorous adjudication process for identifying cardiovascular events, this cohort has been used to describe incident stroke [9] and incident myocardial infarction [10]. In light of shared risk factors between these conditions and HF, the REGARDS cohort is particularly well-suited for describing incident HF as well. Given its racial and geographic diversity, large sample size, extensive covariate assessment, and longitudinal follow-up, the REGARDS cohort has the potential to provide generalizable insights on incident HF. In order to distinguish incident from prevalent HF, it is necessary to assemble a cohort free of HF at baseline. Accordingly, we aimed to develop and validate a simple medication-based strategy for assembling a HF-free cohort within REGARDS.

## Methods

### Study population

The REasons for Geographic And Racial Differences in Stroke (REGARDS) study includes 30,239 African American and white men and women recruited from the 48 contiguous United States between 2003 and 2007 [9]. The REGARDS cohort was devised to study antecedents of racial and geographic differences in stroke mortality in the United States. Participants were community-dwellers who self-identified as white or African American and had to be age ≥ 45 years at enrollment. Participants were enrolled in 2003–2007 using a stratified random sampling approach that balanced race, sex, and geographic location, with planned oversampling of the Southeastern United States, where the burden of cardiovascular disease is high. All participants completed a 45-minute baseline telephone interview ascertaining details of medical history, followed by an in-home visit collecting blood and urine samples as well as physiologic data (blood pressure, height and weight, electrocardiogram). REGARDS participants are additionally linked to Medicare claims data using Social Security numbers, sex, and date of birth as previously described [11]. All REGARDS participants provided written informed consent and signed medical record release forms allowing REGARDS investigators to retrieve medical records for research purposes. The Institutional Review Boards of the University of Alabama at Birmingham and Weill Cornell Medicine approved this research.

### Baseline REGARDS data collection

REGARDS participants completed a computer-assisted telephone interview which collected information on sociodemographics and medical history. Following the interview, a trained health technician performed an in-home examination to collect blood and urine specimens, measure blood pressure, conduct an electrocardiogram, and record an inventory of medications used by the participants. Age, race, sex, need to sleep on multiple pillows (orthopnea), and waking at night because of breathlessness (paroxysmal nocturnal dyspnea; PND) were self-reported. Atrial fibrillation was assessed based on self-reported diagnosis or atrial fibrillation recorded during the electrocardiogram. Hypertension was assessed based on self-reported use of medications to control blood pressure or measured blood pressure greater than 140 mmHg systolic or 90 mmHg diastolic.

### Assembly of a HF-free cohort within REGARDS

**Fig. 1:**
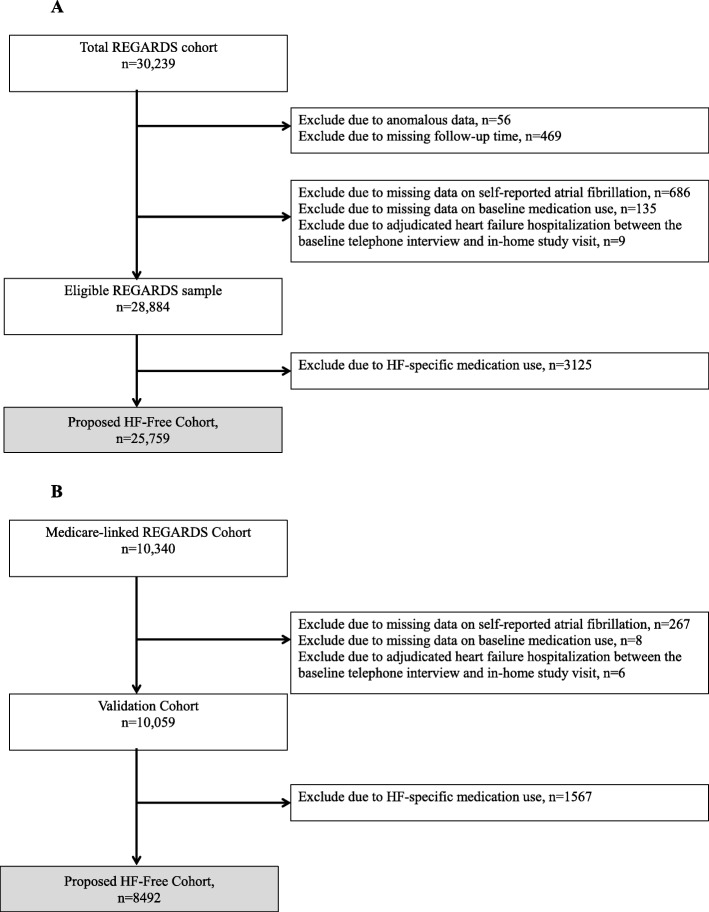
Exclusion cascade to assemble a heart failure-free cohort. First, we excluded those with anomalous data and those with missing follow-up. We then sequentially excluded suspected heart failure by excluding participants HF-specific medications (any of the following: digoxin in the absence of atrial fibrillation; angiotensin-converting enzyme inhibitor/angiotensin receptor blocker plus beta-blocker in absence of hypertension; carvedilol; spironolactone; loop diuretics including furosemide, bumetanide, or torsemide; and/or combination of hydralazine and nitrates). Abbreviations: *AF* Atrial fibrillation, *HF* Heart failure

Among the 30,239 eligible participants in REGARDS, we excluded 56 participants with anomalous data and 469 with no follow-up beyond the baseline assessment. We also excluded 686 participants with missing data on self-reported atrial fibrillation, 135 participants with missing data on baseline medication use, and 9 participants who had adjudicated HF hospitalizations between the baseline telephone interview and the in-home study visit. The remaining cohort included 28,884 eligible participants (Fig. 1a). To assemble a HF-free cohort within REGARDS, we developed a medication-based exclusion cascade, excluding participants with suspected HF using REGARDS study variables that indicated potential treatment for HF. As shown in Fig. 1, we excluded suspected HF by excluding participants taking HF-specific medications (any of the following: digoxin in the absence of atrial fibrillation, angiotensin-converting enzyme inhibitor/angiotensin receptor blocker plus beta-blocker in the absence of hypertension; carvedilol; spironolactone; loop diuretics including furosemide, bumetanide, or torsemide; and/or a combination of hydralazine and nitrates).

### HF hospitalizations among the REGARDS study sample

REGARDS participants were followed through telephone calls twice per year. Participants were asked to report if they had been hospitalized and if so, the reason for hospitalization. Medical records were retrieved for hospitalizations suspected to be associated with select conditions including HF. Medical records were independently reviewed and adjudicated by two clinician-investigators and disagreements were resolved by committee. Adjudicators determined whether HF was present during the hospitalization based on signs and symptoms of HF, imaging findings, and biomarkers such as b-type natriuretic peptide.

### Validation cohort

To validate our medication-based assembly of a HF-free cohort, we identified a subset of REGARDS participants who had at least six months of available Medicare fee-for-service inpatient and outpatient claims data at the time of the baseline in-home study examination (*n* = 10,340) [11]. Medicare is a federal health insurance program in the United States that covers inpatient and outpatient care for adults aged 65 and older and younger individuals with disabilities or end-stage renal disease. It is estimated that about 80% of adults with HF in the United States are Medicare beneficiaries [12]. The Medicare program is funded through income tax, premiums paid by Medicare beneficiaries, and the federal budget. Individuals can opt for fee-for-service coverage which requires copays or privately managed care with a variety of fee structures. Because claims for the privately managed programs were not available, this study is limited to individuals with fee-for-service coverage at the time of study entry. We additionally excluded 267 participants with missing data on self-reported atrial fibrillation, 8 participants with missing data on baseline medication use, and 6 participants who had adjudicated HF hospitalizations between the baseline telephone interview and the in-home study visit. The remaining cohort included 10,059 eligible participants (Fig. 1b).

### Referent definitions of HF for evaluation

To assess the validity of our approach to assemble a HF-free cohort, we created 3 Medicare claims-based definitions of HF to serve as referent comparators. First, we defined a HF hospitalization as at least one inpatient claim for a principal diagnosis of HF (“Hospitalization for HF”). Secondly, we defined a principal diagnosis of HF as at least one inpatient claim with a principal discharge diagnosis of HF or at least two claims for outpatient physician visits (including emergency department visits) with principal diagnoses of HF (“Principal diagnosis of HF”). Finally, we defined “any diagnosis” of HF as at least one inpatient claim or two claims for outpatient physician visits where HF was a principal or secondary diagnosis (“Any diagnosis of HF”). During the period when REGARDS participants were enrolled (2003–2007), the United States used the *International Classification of Diseases*, 9th revision, Clinical Modification (ICD-9-CM). We defined HF according to ICD-9-CM diagnosis codes 402.01, 402.11, 402.91, 404.01, 404.03, 404.11, 404.13, 404.91, 404.93, and 428.xx, as used by the Centers for Medicare & Medicaid Services for publicly reporting HF quality measures [13]. Claims-based definitions of HF were based on all available Medicare claims from prior to the in-home visit date (minimum of 6 months).

### Statistical analysis

We first compared the characteristics of participants included and excluded from the HF-free cohort using means and standard deviations for continuous variables and numbers and percentages for categorical variables. Among the population with linked Medicare claims, we calculated negative predictive value using each of the 3 Medicare claims-based definitions of HF as the referent. The negative predictive value represents the proportion of the HF-free cohort who did not have evidence of HF based on Medicare claims, and is thus the most important diagnostic performance parameter for assembling a HF-free cohort. We also calculated sensitivity, specificity, and positive predictive values for each of the 3 Medicare claims-based definitions. We calculated 95% confidence intervals using the normal approximation. In addition, we examined the performance of the HF-free cohort stratified by subgroups chosen a priori. These subgroups were based on age (≥75 versus 65–75 years of age), sex, and race. Analyses were conducted using SAS 9.4 (Cary, NC).

We also conducted sensitivity analyses to determine the incremental value of additionally excluding participants with self-reported orthopnea and/or PND from the HF-free cohort, as well as the incremental value of additionally excluding participants with adjudicated HF hospitalization within one year of follow-up.

## Results

### Study population

The study sample was comprised of 28,884 eligible REGARDS participants. Among them, 25,759 participants (89%) did not take HF-specific medications and were therefore included in the HF-free cohort. Characteristics of the HF-free cohort and the 3125 who were excluded due to suspected HF are shown in Table 1A. Notably, participants excluded due to suspected HF had a higher prevalence of several comorbid conditions including coronary artery disease, myocardial infarction, atrial fibrillation, hypertension, and diabetes compared to participants in the HF-free cohort. The HF hospitalization rate for the entire study sample was 6.29 per 1000 person-years (Table 2). Events were 6-fold more likely among individuals who were excluded (26.6 per 1000 person-years) compared to those included in the HF-free cohort (4.29 per 1000 person-years).

**Table 1:** Characteristics of REGARDS participants

A. Among eligible REGARDS participants			
	Eligible REGARDS Participants	HF-Free Cohort	Suspected HF
Number	28,884	25,759	3125
Age, mean (SD)	64.9 (9.4)	64.5 (9.3)	68.4 (9.3)
Female Sex	15,875 (55.0%)	14,161 (55.0%)	1714 (54.9%)
African American	11,823 (40.9%)	10,295 (40.0%)	1528 (48.9%)
BMI, mean (SD)	29.3 (6.2)	28.9 (5.9)	32.3 (7.4)
Coronary artery disease^a^	5040 (17.6%)	3711 (14.5%)	1329 (43.2%)
Myocardial infarction^a^	3571 (12.5%)	2605 (10.2%)	966 (31.5%)
Atrial fibrillation	2540 (8.8%)	1894 (7.4%)	646 (20.7%)
Stroke^a^	1813 (6.3%)	1374 (5.4%)	439 (14.1%)
Diabetes^a^	6078 (21.8%)	4684 (18.9%)	1394 (46.3%)
ACR ≥ 30^a^	4154 (15.1%)	3318 (13.5%)	836 (28.6%)
eGFR < 60^a^	3139 (11.3%)	2277 (9.2%)	862 (29.4%)
Hypertension	17,101 (59.2%)	14,704 (57.1%)	2397 (76.7%)
Dyslipidemia^a^	16,495 (59.3%)	14,358 (57.8%)	2137 (71.3%)
Chronic lung disease	2656 (9.2%)	2159 (8.4%)	497 (15.9%)
B. Among Medicare based Validation Cohort			
	Validation Cohort	HF-Free Cohort	Suspected HF
Number	10,059	8492	1567
Age, mean (SD)	71.4 (7.3)	71.3 (7.2)	71.6 (8.1)
Female Sex	5160 (51.3%)	4355 (51.3%)	805 (51.4%)
African American	3519 (35.0%)	2842 (33.5%)	677 (43.2%)
BMI, mean (SD)	28.7 (5.9)	28.2 (5.5)	31.4 (7.0)
Coronary artery disease	2517 (25.0%)	1764 (20.8%)	753 (48.1%)
Myocardial infarction	1693 (16.8%)	1169 (13.8%)	524 (33.4%)
Atrial fibrillation	1144 (11.4%)	792 (9.3%)	352 (22.5%)
Stroke	904 (9.0%)	658 (7.7%)	246 (15.7%)
Diabetes	2407 (23.9%)	1713 (20.2%)	694 (44.3%)
ACR ≥ 30	1767 (17.6%)	1327 (15.6%)	440 (28.1%)
eGFR < 60	2213 (22%)	1628 (19.2%)	585 (37.3%)
Hypertension	6652 (66.1%)	5458 (64.3%)	1194 (76.2%)
Dyslipidemia	6147 (61.1%)	5069 (59.7%)	1078 (68.8%)
Chronic lung disease	1100 (10.9%)	834 (9.8%)	266 (17.0%)

^a^Percentages were determined after excluding participants with missing data for the given variable

Abbreviations

*ACR* Albumin-to-creatinine ratio

*BMI* Body mass index

*eGFR* Estimated glomerular filtration rate

*HF* Heart failure

*REGARDS* REasons for Geographic And Racial Differences in Stroke

*SD* Standard deviation

**Table 2:** Heart failure hospitalization rates in REGARDS participants

	Eligible REGARDS Sample	HF-Free Cohort	Suspected HF
Number of participants	28,884	25,759	3125
Heart Failure Hospitalizations, N	1375	855	520
Rate per 1000 person-years (95% Confidence Interval)	6.29 (5.96, 6.62)	4.29 (4.01, 4.58)	26.6 (24.3, 28.9)

Abbreviations

*REGARDS* REasons for Geographic And Racial Differences in Stroke

*HF* Heart failure

### Medicare-based validation of HF-free cohort

The validation cohort was comprised of 10,059 eligible REGARDS participants. Compared to the broader REGARDS cohort, patients in the validation cohort were older and had a slightly higher prevalence of several comorbid conditions. Among the validation cohort, 8492 participants (84%) did not take HF-specific medications and were therefore included in the HF-free cohort. Characteristics of the HF-free cohort and those who were excluded due to suspected HF based on diagnosis codes are shown in Table 1B. Similar to the observation in the broader study population, participants excluded due to suspected HF had a higher prevalence of coronary artery disease, myocardial infarction, atrial fibrillation, hypertension and diabetes compared to participants in the HF-free cohort.

Depending on the diagnostic-code definition used as the referent, the negative predictive value of the medication based approach to identifying a HF-free cohort ranged from 95.0–99.2% (Table 3). The negative predictive value represents the proportion of the HF-free cohort who did not have evidence of HF based on Medicare claims. Using the most stringent referent definition (Medicare claims for hospitalization with HF as the principal diagnosis), 0.8% of the HF-free cohort had evidence of a history of HF at baseline. Using the least stringent definition (Medicare claims for inpatient or outpatient care with a HF diagnosis), 5% of the HF-free cohort had evidence of a HF history at baseline (3.8% with a principal diagnosis and 1.2% with a secondary diagnosis). This translated into the inclusion of just 64 to 426 participants with a Medicare-based diagnosis of HF (depending on the referent used) into the HF-free cohort.

**Table 3:** Diagnostic performance (95% confidence intervals) of heart failure-free cohort compared to Medicare referent standards

		HF according to Medicare N (%)	Excluded from HF-free cohort N	Included in HF-free cohort N	NPV %	PPV %	Sens %	Spec %
Hospitalization for HF	**+**	293 (2.9%)	229	64	99.2% (99.1–99.4%)	14.6% (12.9–16.4%)	78.2% (73.4–82.9%)	86.3% (85.6–87.0%)(
	**–**	9766 (97.1%)	1338	8428				
Principal diagnosis of HF	**+**	968 (9.6%)	642	326	96.2% (95.8–96.6%)	41.0% (38.5–43.4%)	66.3% (63.3–69.3%)	89.8% (89.2–90.4%)
	**–**	9091 (90.4%)	925	8166				
Any diagnosis of HF	**+**	1135 (11.3%)	709	426	95.0% (94.5–95.4%)	45.2% (42.8–47.7%)	62.5% (59.6–65.3%)	90.4% (89.8–91.0%)
	**–**	8924 (88.7%)	858	8066				

Abbreviations

*HF* Heart failure

*NPV* Negative predictive value

*PPV* Positive predictive value

*Sens* Sensitivity

*Spec* Specificity

Specificity ranged from 86.3–90.4%, and sensitivity ranged from 62.5–78.2% (Table 3). The number of participants without a Medicare-based diagnosis of HF excluded from our cohort ranged from 858 to 1338 depending on referent used. Notably, performance was stable across age, sex, and race strata (Additional files 1, 2, and 3).

### Sensitivity analysis

The incremental value of additionally excluding self-reported symptoms (orthopnea and/or PND) to assemble a HF-free cohort was limited; negative predictive value improved slightly, but at a sacrifice of excluding an additional thousand participants (Table 4A). Similarly, additionally excluding adjudicated HF hospitalizations within one year of follow-up provided little incremental improvement to negative predictive value (Table 4B).

**Table 4:** Sensitivity analysis for diagnostic performance (95% confidence intervals) compared to Medicare referent standards

A. With addition of self-reported symptoms (orthopnea and/or paroxysmal nocturnal dyspnea)								
		HF according to Medicare N (%)	Excluded from HF-free cohort N	Included in HF-free cohort N	NPV %	PPV %	Sens %	Spec %
Hospitalization for HF	**+**	293 (2.9%)	256	37	99.5% (99.3–99.7%)	9.5% (8.4–10.6%)	87.4% (83.6–91.2%)	75.1% (74.2–75.9%)
	**–**	9766 (97.1%)	2436	7330				
Principal diagnosis of HF	**+**	968 (9.6%)	746	222	97.0% (96.6–97.4%)	27.7% (26.0–29.4%)	77.1% (74.4–79.7%)	78.6% (77.8–79.4%)
	**–**	9091 (90.4%)	1946	7145				
Any diagnosis of HF	**+**	1135 (11.3%)	838	297	96.0% (95.5–96.4%)	31.1% (29.4–32.9%)	73.8% (71.3–76.4%)	79.2% (78.4–80.1%)
	**–**	8924 (88.7%)	1854	7070				
B. With addition of adjudicated heart failure								
		HF according to Medicare N (%)	Excluded from HF-free cohort N	Included in HF-free cohort N	NPV %	PPV %	Sens %	Spec %
Hospitalization for HF	**+**	293 (2.9%)	236	57	99.3% (99.2–99.5%)	14.6% (12.9–16.4%)	80.5% (76.0–85.1%)	85.9% (85.2–86.6%)
	**–**	9766 (97.1%)	1375	8391				
Principal diagnosis of HF	**+**	968 (9.6%)	657	311	96.3% (95.9–96.7%)	40.8% (38.4–43.2%)	67.9% (64.9–70.8%)	89.5% (88.9–90.1%)
	**–**	9091 (90.4%)	954	8137				
Any diagnosis of HF	**+**	1135 (11.3%)	724	411	95.1% (94.7–95.6%)	44.9% (42.5–47.4%)	63.8% (61.0–66.6%)	90.1% (89.4–90.7%)
	**–**	8924 (88.7%)	887	8037				

## Discussion

By excluding participants based on their use of HF-specific medications, we successfully assembled a cohort within REGARDS where very few participants had a history of HF. This HF-free cohort can be examined in the future to study incident HF among a contemporary geographically-diverse study sample. Notably, HF hospitalization rate for the HF-free cohort was one-sixth the rate for individuals excluded due to possible baseline HF. The HF hospitalization rate for the HF-free cohort was much lower than the rates observed in major HF trials, lending additional support for our approach of identifying a cohort free of HF [14]. We further validated our approach using Medicare claims data as the referent, yielding excellent negative predictive value; negative predictive value remained above 95% regardless of the referent comparator.

Prior observational studies of HF-free cohorts used to identify and describe incident HF have included those derived from communities in Olmstead County, Minnesota [2] and Framingham, Massachusetts, [3] as well as multi-community cohorts from the CHS, [6] Health ABC, [7] and ARIC [8]. These studies have developed HF-free cohorts by detecting and excluding HF based on self-report, in some cases followed by medical record review. The approach of identifying HF via self-report may be limited by suboptimal negative predictive value (86% in ARIC based on reported prevalence of 18.6%, sensitivity of 33% and specificity of 97%) [15] and suboptimal reliability with reported kappa statistics as low as 0.44 [16]. Our strategy of utilizing HF-specific medications to identify and exclude participants with HF obviated dependence on the self-reporting of HF, a substantial source of bias, and provided a practical method for assembling a HF-free cohort. This approach was particularly well-suited for the REGARDS study sample, in which medication history was collected at baseline via medication inventory, and was available in nearly 100% of the study sample.

Given the large number of pharmacologic agents that are indicated for use in HF, many of which are specific to HF, [17, 18] we speculated that leveraging HF medication use to identify (and subsequently exclude) participants with HF would provide a robust strategy for assembling a HF-free cohort. Given its excellent specificity for HF, [19] we incorporated the use of digoxin in the absence of atrial fibrillation to identify and exclude participants from our HF-free cohort. To further capitalize on the concept of HF medication use in the absence of other (non-HF) indications, we also incorporated the use of neuro-hormonal blockers (renin-angiotensin inhibitors and beta blockers) in the absence of hypertension to identify and exclude participants from our HF-free cohort. Finally, we incorporated medications specific for HF that lacked other (non-HF) common guideline-based indications to identify participants with HF—we subsequently identified and excluded participants taking carvedilol which is a beta-blocker specifically indicated for HF, spironolactone which is an aldosterone inhibitor indicated for HF (of note, eplerenone use was exceedingly rare in this cohort), loop diuretics which are the preferred agents for decongestion, [17] and pre-specified combinations of vasodilators (hydralazine and nitrates) indicated for use in HF.

Our approach of excluding participants with suspected HF based on their medication use optimized negative predictive value, the diagnostic performance parameter most important to assembling a HF-free cohort. In developing our strategy to assemble a HF-free cohort, we also considered the potential consequence of excessively excluding participants, which would limit the size and generalizability of the cohort, and could significantly hamper statistical power for future studies. Accordingly, our approach preserved a moderately-sized cohort of 25,759 participants. In sum, our strategy maximized negative predictive value without significantly sacrificing the size of the cohort.

HF affects a diverse population that includes varying age, men and women, and whites and African Americans [1, 20]. Prior studies have shown that prescribing patterns for HF may differ based on age, [21] sex, [22] and race [23]. Accordingly, we examined the diagnostic performance of our medication-based strategy to assemble a HF-free cohort among age-, sex-, and race-related subgroups chosen a priori. Our findings revealed that performance was stable across both age strata, men and women, and whites and African Americans. This supports the use of our strategy to assemble a HF-free cohort, without evidence of bias related to age, sex, or race.

When screening for HF, symptomatology like orthopnea and PND are frequently assessed. We therefore examined the impact of incorporating these self-reported symptoms in our medication-based strategy. Our results revealed that orthopnea and/or PND did not substantively improve negative predictive value. This is consistent with limitations of performance observed for the diagnosis of HF in the clinical context [24]. Thus, we concluded that, to identify and exclude participants with HF, our simple medication-based strategy did not benefit from the addition of self-reported symptoms.

There were some limitations to our study that should be noted. Sensitivity and positive predictive value were not particularly high; thus this strategy may be more appropriate for developing a HF-free cohort than for detecting HF. Although a medication inventory was collected for nearly all REGARDS participants, we cannot be certain that these were complete in all cases. Finally, Medicare is an imperfect referent due to limitations in the accuracy of claims-based diagnoses [25]. Further, our study excluded REGARDS participants without Medicare, and we are unable to draw definitive conclusions about younger individuals with HF. Participants with available Medicare claims at baseline were older and had a higher prevalence of chronic medical conditions compared to the broader REGARDS study population.

## Conclusion

Our investigation has assembled and validated a HF-free cohort in REGARDS, which can serve as the basis for future studies to examine incident HF in REGARDS and perhaps similar study samples. Our medication-based approach may be worth examining in other cohorts that have rigorous methods of data collection on medications to ascertain its generalizability. REGARDS in particular offers an excellent cohort for studying incident HF because it is a contemporary geographically diverse cohort with a large number of African American and white participants. Accordingly, through this HF-free cohort, REGARDS has the potential to provide insights on incident HF.

## Supplementary information

**Additional file 1.** Performance of approach to assemble a heart failure-free cohort in the REasons for Geographic And Racial Differences in Stroke (REGARDS) study population compared to Medicare referent standards, according to age

**Additional file 2.** Performance of approach to assemble a heart failure-free cohort in the REasons for Geographic And Racial Differences in Stroke (REGARDS) study population compared to Medicare referent standards, according to sex

**Additional file 3.** Performance of approach to assemble a heart failure-free cohort in the REasons for Geographic And Racial Differences in Stroke (REGARDS) study population compared to Medicare referent standards, according to race

## Data Availability

The datasets generated and/or analyzed during the current study are not publicly available, as the data includes characteristics that may compromise individual patient privacy; but are available from the corresponding author on reasonable request.

## Abbreviations

ARIC: Atherosclerosis risk in communities study
CHS: Cardiovascular health study
Health ABC: Dynamics of health, aging, and body composition study
HF: Heart failure
ICD-9-CM: International classification of diseases, 9th revision, clinical modification diagnosis codes (ICD-9-CM)
PND: Paroxysmal nocturnal dyspnea
REGARDS: Reasons for geographic and racial differences in stroke

## References

[1] Mozaffarian D, Benjamin EJ, Go AS, Arnett DK, Blaha MJ, Cushman M (2015). Heart disease and stroke statistics--2015 update: a report from the American Heart Association. Circ.

[2] Roger VL, Weston SA, Redfield MM, Hellermann-Homan JP, Killian J, Yawn BP (2004). Trends in heart failure incidence and survival in a community-based population. JAMA.

[3] Kannel WB, D'Agostino RB, Silbershatz H, Belanger AJ, Wilson PW, Levy D (1999). Profile for estimating risk of heart failure. Arch Intern Med.

[4] Masoudi FA, Havranek EP, Wolfe P, Gross CP, Rathore SS, Steiner JF (2003). Most hospitalized older persons do not meet the enrollment criteria for clinical trials in heart failure. Am Heart J.

[5] Cherubini A, Oristrell J, Pla X, Ruggiero C, Ferretti R, Diestre G (2011). The persistent exclusion of older patients from ongoing clinical trials regarding heart failure. Arch Intern Med.

[6] Gottdiener JS, Arnold AM, Aurigemma GP, Polak JF, Tracy RP, Kitzman DW (2000). Predictors of congestive heart failure in the elderly: the cardiovascular health study. J Am Coll Cardiol.

[7] Kalogeropoulos A, Georgiopoulou V, Kritchevsky SB, Psaty BM, Smith NL, Newman AB (2009). Epidemiology of incident heart failure in a contemporary elderly cohort: the health, aging, and body composition study. Arch Intern Med.

[8] Chang PP, Chambless LE, Shahar E, Bertoni AG, Russell SD, Ni H (2014). Incidence and survival of hospitalized acute decompensated heart failure in four US communities (from the atherosclerosis risk in communities study). Am J Cardiol.

[9] Howard VJ, Cushman M, Pulley L, Gomez CR, Go RC, Prineas RJ (2005). The reasons for geographic and racial differences in stroke study: objectives and design. Neuroepidemiology.

[10] Soliman EZ, Safford MM, Muntner P, Khodneva Y, Dawood FZ, Zakai NA (2014). Atrial fibrillation and the risk of myocardial infarction. JAMA Intern Med.

[11] Xie F, Colantonio LD, Curtis JR, Safford MM, Levitan EB, Howard G (2016). Linkage of a population-based cohort with primary data collection to Medicare claims: the reasons for geographic and racial differences in stroke study. Am J Epidemiol.

[12] Blecker S, Paul M, Taksler G, Ogedegbe G, Katz S (2013). Heart failure-associated hospitalizations in the United States. J Am Coll Cardiol.

[13] International Classification of Diseases,Ninth Revision, Clinical Modification (ICD-9-CM): Centers for Disease Control and Prevention; 2013 [Available from: http://www.cdc.gov/nchs/icd/icd9cm.htm

[14] Campbell RT, Jhund PS, Castagno D, Hawkins NM, Petrie MC, McMurray JJ (2012). What have we learned about patients with heart failure and preserved ejection fraction from DIG-PEF, CHARM-preserved, and I-PRESERVE?. J Am Coll Cardiol.

[15] Camplain R, Kucharska-Newton A, Loehr L, Keyserling TC, Layton JB, Wruck L (2017). Accuracy of self-reported heart failure. The atherosclerosis risk in communities (ARIC) study. J Card Fail.

[16] Langer RD, White E, Lewis CE, Kotchen JM, Hendrix SL, Trevisan M (2003). The Women's Health Initiative observational study: baseline characteristics of participants and reliability of baseline measures. Ann Epidemiol.

[17] Yancy CW, Jessup M, Bozkurt B, Butler J, Casey DE, Drazner MH (2013). 2013 ACCF/AHA guideline for the management of heart failure: executive summary: a report of the American College of Cardiology Foundation/American Heart Association task force on practice guidelines. Circ.

[18] Yancy CW, Jessup M, Bozkurt B, Butler J, Casey DE, Colvin MM (2016). 2016 ACC/AHA/HFSA focused update on new pharmacological therapy for heart failure: an update of the 2013 ACCF/AHA guideline for the Management of Heart Failure: a report of the American College of Cardiology/American Heart Association task force on clinical practice guidelines and the Heart Failure Society of America. J Am Coll Cardiol.

[19] Fonseca C, Oliveira AG, Mota T, Matias F, Morais H, Costa C (2004). Evaluation of the performance and concordance of clinical questionnaires for the diagnosis of heart failure in primary care. Eur J Heart Fail.

[20] Goyal P, Almarzooq ZI, Horn EM, Karas MG, Sobol I, Swaminathan RV (2016). Characteristics of Hospitalizations for Heart Failure with Preserved Ejection Fraction. Am J Med.

[21] Lee DS, Tu JV, Juurlink DN, Alter DA, Ko DT, Austin PC (2005). Risk-treatment mismatch in the pharmacotherapy of heart failure. JAMA.

[22] Rathore SS, Foody JM, Wang Y, Herrin J, Masoudi FA, Havranek EP (2005). Sex, quality of care, and outcomes of elderly patients hospitalized with heart failure: findings from the National Heart Failure Project. Am Heart J.

[23] Yancy CW, Abraham WT, Albert NM, Clare R, Stough WG, Gheorghiade M (2008). Quality of care of and outcomes for African American hospitalized with heart failure: findings from the OPTIMIZE-HF (organized program to initiate lifesaving treatment in hospitalized patients with heart failure) registry. J Am Coll Cardiol.

[24] Wang CS, FitzGerald JM, Schulzer M, Mak E, Ayas NT (2005). Does this dyspneic patient in the emergency department have congestive heart failure?. JAMA.

[25] Kucharska-Newton AM, Heiss G, Ni H, Stearns SC, Puccinelli-Ortega N, Wruck LM (2016). Identification of heart failure events in Medicare claims: the atherosclerosis risk in communities (ARIC) study. J Card Fail.

