# Supplementary material for: Assembling and validating a heart failure-free cohort from the Reasons for Geographic and Racial Differences in Stroke (REGARDS) study
Source: BMC Med Res Methodol. 2020 Mar 4;20:53. doi: 10.1186/s12874-019-0890-x (PMC7055019; doi:10.1186/s12874-019-0890-x)
Supplement: Supplementary file 1 — Additional file 1. Performance of approach to assemble a heart failure-free cohort in the REasons for Geographic And Racial Differences in Stroke (REGARDS) study population compared to Medicare referent standards, according to age [file 12874_2019_890_MOESM1_ESM.docx]

**Additional Table 1.** Diagnostic performance (95% confidence interval) of heart failure-free cohort compared to Medicare referent standards, according to age

1. **Age**≥**75 years**

|  |  | **HF according to Medicare**  **N (%)** | **Excluded from**  **HF-free cohort**  **N** | **Included in**  **HF-free cohort**  **N** | **NPV**  **%** | **PPV**  **%** | **Sens**  **%** | **Spec**  **%** |
| --- | --- | --- | --- | --- | --- | --- | --- | --- |
| **Hospitalization for HF** | **+** | 117 (3.6%) | 99 | 18 | 99.3%   (99.0-99.6%) | 16.7%   (13.7-19.7%) | 84.6%   (78.1-91.2%) | 84.3%  (83.1-85.6%) |
|  | **-** | 3162 (96.4%) | 495 | 2667 |  |  |  |  |
| **Principal diagnosis of HF** | **+** | 437 (13.3%) | 292 | 145 | 94.6%   (93.7-95.5%) | 49.2%   (45.1-53.2%) | 66.8%  (62.4-71.2%) | 89.4%   (88.2-90.5%) |
|  | **-** | 2842 (86.7%) | 302 | 2540 |  |  |  |  |
| **Any diagnosis of HF** | **+** | 506 (15.4%) | 320 | 186 | 93.1%   (92.1-94.0%) | 53.9%   (49.9-57.9%) | 63.2%   (59.0-67.4%) | 90.1%  (89.0-91.2%) |
|  | **-** | 2773 (84.6%) | 274 | 2499 |  |  |  |  |

Abbreviations:

HF: Heart failure

NPV: Negative predictive value

PPV: Positive predictive value

Sens: Sensitivity

Spec: Specificity

**B**. **Age 65-74 years**

|  |  | **HF according to Medicare**  **N (%)** | **Excluded from**  **HF-free cohort**  **N** | **Included in**  **HF-free cohort**  **N** | **NPV**  **%** | **PPV**  **%** | **Sens**  **%** | **Spec**  **%** |
| --- | --- | --- | --- | --- | --- | --- | --- | --- |
| **Hospitalization for HF** | **+** | 176 (2.6%) | 130 | 46 | 99.2%   (99.0-99.4%) | 13.4%  (11.2-15.5%) | 73.9%   (67.4-80.4%) | 87.2%  (86.4-88.0%) |
|  | **-** | 6604 (97.4%) | 843 | 5761 |  |  |  |  |
| **Principal diagnosis of HF** | **+** | 531 (7.8%) | 350 | 181 | 96.9%  (96.4-97.3%) | 36.0%  (33.0-39.0%) | 65.9%  (61.9-69.9%) | 90.0%   (89.3-90.8%) |
|  | **-** | 6249 (92.2%) | 623 | 5626 |  |  |  |  |
| **Any diagnosis of HF** | **+** | 629 (9.3%) | 389 | 240 | 95.9%   (95.4-96.4%) | 40.0%   (36.9-43.1%) | 61.8%   (58.0-65.6%) | 90.5%  (89.8-91.2%) |
|  | **-** | 6151 (90.7%) | 584 | 5567 |  |  |  |  |

Abbreviations:

HF: Heart failure

NPV: Negative predictive value

PPV: Positive predictive value

Sens: Sensitivity

Spec: Specificity
