# Supplementary material for: Assembling and validating a heart failure-free cohort from the Reasons for Geographic and Racial Differences in Stroke (REGARDS) study
Source: BMC Med Res Methodol. 2020 Mar 4;20:53. doi: 10.1186/s12874-019-0890-x (PMC7055019; doi:10.1186/s12874-019-0890-x)
Supplement: Supplementary file 2 — Additional file 2. Performance of approach to assemble a heart failure-free cohort in the REasons for Geographic And Racial Differences in Stroke (REGARDS) study population compared to Medicare referent standards, according to sex [file 12874_2019_890_MOESM2_ESM.docx]

**Additional Table 2.** Diagnostic performance (95% confidence interval) of heart failure-free cohort compared to Medicare referent standards, according to sex

**A. Men**

|  |  | **HF according to Medicare**  **N (%)** | **Excluded from**  **HF-free cohort**  **N** | **Included in**  **HF-free cohort**  **N** | **NPV**  **%** | **PPV**  **%** | **Sens**  **%** | **Spec**  **%** |
| --- | --- | --- | --- | --- | --- | --- | --- | --- |
| **Hospitalization for HF** | **+** | 149 (3%) | 118 | 31 | 99.3%  (99.0-99.5%) | 15.5%   (12.9-18.1%) | 79.2%   (72.7-85.7%) | 86.4%   (85.5-87.4%) |
|  | **-** | 4750 (97%) | 644 | 4106 |  |  |  |  |
| **Principal diagnosis of HF** | **+** | 497 (10.1%) | 344 | 153 | 96.3%  (95.7-96.9%) | 45.1%  (41.6-48.7%) | 69.2%  (65.2-73.3%) | 90.5%  (89.6-91.4%) |
|  | **-** | 4402 (89.9%) | 418 | 3984 |  |  |  |  |
| **Any diagnosis of HF** | **+** | 573 (11.7%) | 373 | 200 | 95.2%  (94.5-95.8%) | 49.0%  (45.4-52.5%) | 65.1%   (61.2-69.0%) | 91.0%  (90.2-91.9%) |
|  | **-** | 4326 (88.3%) | 389 | 3937 |  |  |  |  |

Abbreviations:

HF: Heart failure

NPV: Negative predictive value

PPV: Positive predictive value

Sens: Sensitivity

Spec: Specificity

**B. Women**

|  |  | **HF according to Medicare**  **N (%)** | **Excluded from**  **HF-free cohort**  **N** | **Included in**  **HF-free cohort**  **N** | **NPV**  **%** | **PPV**  **%** | **Sens**  **%** | **Spec**  **%** |
| --- | --- | --- | --- | --- | --- | --- | --- | --- |
| **Hospitalization for HF** | **+** | 144 (2.8%) | 111 | 33 | 99.2%  (99.0-99.5%) | 13.8%  (11.4-16.2%) | 77.1%  (70.2-83.9%) | 86.2%  (85.2-87.1%) |
|  | **-** | 5016 (97.2%) | 694 | 4322 |  |  |  |  |
| **Principal diagnosis of HF** | **+** | 471 (9.1%) | 298 | 173 | 96.0%  (95.4-96.6%) | 37.0%  (33.7-40.4%) | 63.3%  (58.9-67.6%) | 89.2%  (88.3-90.1%) |
|  | **-** | 4689 (90.9%) | 507 | 4182 |  |  |  |  |
| **Any diagnosis of HF** | **+** | 562 (10.9%) | 336 | 226 | 94.8%  (94.2-95.5%) | 41.7%  (38.3-45.1%) | 59.8%  (55.7-63.8%) | 89.8%  (88.9-90.7%) |
|  | **-** | 4598 (89.1%) | 469 | 4129 |  |  |  |  |

Abbreviations:

HF: Heart failure

NPV: Negative predictive value

PPV: Positive predictive value

Sens: Sensitivity

Spec: Specificity
