# Supplementary material for: Assembling and validating a heart failure-free cohort from the Reasons for Geographic and Racial Differences in Stroke (REGARDS) study
Source: BMC Med Res Methodol. 2020 Mar 4;20:53. doi: 10.1186/s12874-019-0890-x (PMC7055019; doi:10.1186/s12874-019-0890-x)
Supplement: Supplementary file 3 — Additional file 3. Performance of approach to assemble a heart failure-free cohort in the REasons for Geographic And Racial Differences in Stroke (REGARDS) study population compared to Medicare referent standards, according to race [file 12874_2019_890_MOESM3_ESM.docx]

**Additional Table 3.** Diagnostic performance (95% confidence interval) of heart failure-free cohort compared to Medicare referent standards, according to race

1. **Whites**

|  |  | **HF according to Medicare**  **N (%)** | **Excluded from**  **HF-free cohort**  **N** | **Included in**  **HF-free cohort**  **N** | **NPV**  **%** | **PPV**  **%** | **Sens**  **%** | **Spec**  **%** |
| --- | --- | --- | --- | --- | --- | --- | --- | --- |
| **Hospitalization for HF** | **+** | 143 (2.2%) | 118 | 25 | 99.6%  (99.4-99.7%) | 13.3%  (11.0-15.5%) | 82.5%  (76.3-88.7%) | 87.9%  (87.1-88.7%) |
|  | **-** | 6397 (97.8%) | 772 | 5625 |  |  |  |  |
| **Principal diagnosis of HF** | **+** | 526 (8%) | 365 | 161 | 97.2%  (96.7-97.6%) | 41.0%  (37.8-44.2%) | 69.4%  (65.5-73.3%) | 91.3%  (90.6-92.0%) |
|  | **-** | 6014 (92%) | 525 | 5489 |  |  |  |  |
| **Any diagnosis of HF** | **+** | 609 (9.3%) | 398 | 211 | 96.3%  (95.8-96.8%) | 44.7%  (41.5-48.0%) | 65.4%  (61.6-69.1%) | 91.7%  (91.0-92.4%) |
|  | **-** | 5931 (90.7%) | 492 | 5439 |  |  |  |  |

Abbreviations:

HF: Heart failure

NPV: Negative predictive value

PPV: Positive predictive value

Sens: Sensitivity

Spec: Specificity

1. **African Americans**

|  |  | **HF according to Medicare**  **N (%)** | **Excluded from**  **HF-free cohort**  **N** | **Included in**  **HF-free cohort**  **N** | **NPV**  **%** | **PPV**  **%** | **Sens**  **%** | **Spec**  **%** |
| --- | --- | --- | --- | --- | --- | --- | --- | --- |
| **Hospitalization for HF** | **+** | 150 (4.3%) | 111 | 39 | 98.6%  (98.2-99.1%) | 16.4%  (13.6-19.2%) | 74.0%  (67.0-81.0%) | 83.2%  (81.9-84.5%) |
|  | **-** | 3369 (95.7%) | 566 | 2803 |  |  |  |  |
| **Principal diagnosis of HF** | **+** | 442 (12.6%) | 277 | 165 | 94.2%  (93.3-95.1%) | 40.9%  (37.2-44.6%) | 62.7%  (58.2-67.2%) | 87.0%  (85.8-88.2%) |
|  | **-** | 3077 (87.4%) | 400 | 2677 |  |  |  |  |
| **Any diagnosis of HF** | **+** | 526 (14.9%) | 311 | 215 | 92.4%  (91.5-93.4%) | 45.9%  (42.2-49.7%) | 59.1%  (54.9-63.3%) | 87.8%  (86.6-88.9%) |
|  | **-** | 2993 (85.1%) | 366 | 2627 |  |  |  |  |

Abbreviations:

HF: Heart failure

NPV: Negative predictive value

PPV: Positive predictive value

Sens: Sensitivity

Spec: Specificity
